# Understanding the public knowledge and awareness of parkinson’s disease in Ireland: a cross-sectional survey

**DOI:** 10.1186/s12889-025-23573-8

**Published:** 2025-07-03

**Authors:** Sophie Crooks, Gary Mitchell, Lisa Wynne, Gillian Carter

**Affiliations:** 1 School of Nursing and Midwifery, Queen’s University Belfast, Belfast, Northern Ireland UK; 2 Parkinson’s Ireland, Dublin, Ireland

**Keywords:** Parkinson’s disease, Experiences, Awareness, Public perception, Stigma, Public, Healthcare, Quality of life, Public knowledge

## Abstract

**Background:**

Parkinson’s disease (PD) is a progressive neurological disorder characterised by motor and non-motor symptoms that significantly impact quality of life (QoL). Despite its growing prevalence, public awareness and understanding of PD remain limited, contributing to stigma and social isolation. This study evaluates public knowledge and perceptions of PD across Ireland to identify educational gaps and inform public health initiatives.

**Methods:**

A cross-sectional survey was conducted from January to April 2024, using a modified version of existing questionnaires on PD knowledge. The survey was distributed online via social media platforms and charity networks, targeting adults residing in Ireland. Descriptive statistics and chi-square tests were employed to analyse the data. Ethical approval for this study was obtained from the Faculty of Medicine and Health Sciences, Queen’s University Belfast (MHLS23_132).

**Results:**

A total of 796 respondents completed the survey, predominantly female (83.4%) and aged 18–24 (31.7%). While 92.9% recognised PD as a neurological disorder, misconceptions about its classification persisted. Awareness of motor symptoms like tremor and bradykinesia was high, but non-motor symptoms such as chronic fatigue were less recognised. Only 42% were aware of available treatments, and 33.7% had encountered PD-related stigma. Social media emerged as the preferred platform for raising awareness, cited by 46.2% of participants.

**Conclusion:**

The findings reveal critical knowledge gaps and stigma regarding PD, emphasising the need for targeted educational initiatives. Public health campaigns, particularly leveraging social media, are essential to enhance understanding, reduce misconceptions, and improve QoL for individuals with PD. By addressing awareness and encouraging a supportive environment, these initiatives can contribute to better public perception and management of PD.

**Supplementary Information:**

The online version contains supplementary material available at 10.1186/s12889-025-23573-8.

## Background

Parkinson’s disease (PD) is a progressive neurological condition primarily recognised for its motor symptoms, such as tremor, rigidity, and bradykinesia (slowness of movement) [1, 2]. However, PD encompasses a range of both motor and non-motor symptoms, including depression, cognitive impairment, and sleep disturbances, which collectively impact an individual’s quality of life (QoL) [3, 4]. As an aging society continues to grow, the prevalence of PD is increasing, with older adults being the most affected demographic [5]. Despite this growing burden, a significant lack of knowledge and awareness about PD persists within the general public, with misconceptions often shaping perceptions of the disease [6, 7]. This gap in understanding can lead to challenges for individuals with PD, such as social isolation, stigma, and mental health concerns, all of which can further diminish their QoL and well-being [6–11].

In social science literature, there is established evidence linking knowledge, attitudes, perceptions, and misconceptions to health-related behaviours and outcomes [12]. Knowledge, in this context, is defined as the understanding of or information about a subject acquired through experience or study [13]. This acquired knowledge can significantly shape individuals’ attitudes towards a particular issue, often blending their beliefs and experiences into a comprehensive construct referred to as ‘attitude-relevant knowledge’ [14]. In the case of PD, a more informed public can foster attitudes that not only reduce stigma but also contribute to improved health outcomes for people with the disease [6].

Public health is a multidisciplinary field that focuses on improving the health of populations through prevention, education, and policy [15]. It plays a crucial role in addressing the social, economic, and environmental factors that affect the health and well-being of communities [16]. In the context of PD, public health initiatives are essential in raising awareness, improving knowledge, and advocating for the needs of people living with PD [6]. Public health interventions targeting awareness can reduce the stigma associated with PD, encourage early diagnosis and intervention, and promote a more supportive environment for individuals with the condition [7]. These efforts ultimately contribute to improving the overall QoL for those affected by PD.

Despite some studies identifying gaps in public knowledge about PD [6, 17], there is limited research specifically examining the knowledge and perceptions of PD across the island of Ireland. This gap highlights the need for a targeted evaluation of public awareness of PD within this specific region. A UK study [18] has shown that negative attitudes and stigma around PD, stemming from a lack of understanding, can have a serious impact on mental health, contributing to feelings of stress, anxiety, and depression. These effects are often stronger when people with PD internalise stigma or feel judged by others [18]. Understanding the general public’s knowledge, attitudes, and perceptions of PD is essential in identifying areas where education and resources are lacking. By assessing the level of awareness, it becomes possible to develop and implement more effective public health campaigns that can increase understanding and reduce misconceptions about the disease.

## Aim

To explore the public knowledge and understanding of PD across the island of Ireland.

### Objectives

1. To explore the level of public knowledge and understanding of PD across the island of Ireland.
2. To identify demographic patterns and factors associated with varying levels of PD awareness.
3. To determine specific gaps in public knowledge that can inform targeted educational and awareness campaigns.

## Methods

### Ethics

#### Ethical approval

for this study was obtained from the Faculty of Medicine and Health Sciences, Queen’s University Belfast (MHLS23_132). The study did not take place in a clinical setting, therefore did not require NHS ethical approval or specific hospital trust approval. This study was conducted and reported in accordance with the STROBE (Strengthening the Reporting of Observational Studies in Epidemiology) guidelines. A completed STROBE checklist is available in Additional File 1.

An online cross-sectional survey was developed and administered to members of the public in Ireland between January and April 2024.

### Survey tool

The survey used in this study was developed in response to findings from a scoping review [6] which highlighted a questionnaire [19] which set out to investigate public knowledge and awareness of PD in South Korea. The original survey from South Korea was deemed culturally unsuitable for Ireland following engagement with stakeholders, including PI and PD UK. Key issues included language, question framing, and stigma-related content that did not align with Irish cultural perspectives or public attitudes toward PD. To address this, we collaborated with these organisations to develop additional items focused on stigma and awareness that were culturally relevant and tailored to the Irish context. The survey was then adapted and deployed electronically to suit the study’s target population and objectives. The questionnaire items were chosen to assess key areas of public knowledge and perceptions that impact both health outcomes and stigma related to PD. For example, questions about the genetic nature of PD help identify misconceptions that could contribute to fear or stigma within families. Items on treatment awareness and the existence of a cure gauge understanding that influences health-seeking behaviour and realistic expectations. Overall, these questions help pinpoint knowledge gaps and stigma drivers, which are essential for designing effective educational and support interventions. A list of questions for the questionnaire can be seen in Additional File 2.

### Pilot questionnaire

Prior to data collection, the online questionnaire was piloted in December 2023 with a sample of 14 participants. This pilot was conducted to assess the face validity of the questionnaire to ensure the questions appeared to measure what they were intended to and were clear and appropriate for the target audience. Participants were informed that they were taking part in a pilot study only, and none of their responses were included in the final data analysis. Feedback was used to refine the questionnaire and accompanying information sheets. Based on participant suggestions, some items were reordered and reworded for clarity, for example, the term *bradykinesia* was replaced with *slow movement* to improve understanding.

### Sampling procedures for cross-sectional survey

Members of the public were the research participants for this phase of the study. These participants were recruited using convenience sampling due to the recruitment process taking place via social media platforms. Recruitment for the survey was conducted exclusively via social media platforms due to their broad reach, cost-effectiveness, and feasibility. Inclusion and exclusion criteria for this phase guided recruitment (Table 1):

**Table 1:** Inclusion/exclusion criteria for survey participants

Inclusion Criteria	Exclusion Criteria
• Member of the public • Participants must be at least 18 years old • Must live in Ireland (north or south)	• Those under the age of 18 years old • Those not residing in Ireland (north or south)

### Recruitment for cross-sectional survey

Members of the public were recruited as research participants to take part in a cross-sectional survey. Social media platforms, such as Facebook, Instagram and X, were used to disseminate the survey to the wider population of Ireland, including Northern Ireland (NI) and the Republic of Ireland (ROI). The link to the survey was advertised on social media pages of Parkinson’s UK and Parkinson’s Ireland. Age NI and Age Friendly Ireland, two leading charities for older people in NI and ROI, also shared links to the survey on social media pages. These charities also sent emails to members of the charity with links to the survey. The researcher followed university policies and procedures for advertising and recruiting through social media. Posts on social media and emails included a direct link to the survey, allowing easy access for participants. When participants accessed the link, they were taken to the MS Forms survey, where more details of the study were provided and the option to participate in the survey was given. The link was reposted on social media pages twice a week for 8 weeks, then taken down. Studies within a recent scoping review [6] exploring the public perception of PD had sample sizes ranging from 13 to 2069 participants, with the median number being 167. Therefore, the target number of participants this study aimed to recruit was approximately 200–300 participants. Despite this, the total number of participants who completed the online survey was 796 people.

### Analysis

Data was collected and transferred from MS Forms and analysed using Statistical Package for Social Sciences (SPSS) software version 27 [20]. Descriptive statistics provided an overview of respondents’ knowledge levels and perceptions. Chi-square tests investigated associations, such as the relationship between experience levels and awareness of treatments. A p-value of < 0.05 was considered statistically significant. Subgroup analyses by demographics like age and experience was conducted to offer insights into variations within the public’s perceptions and knowledge. This comprehensive analysis was conducted to gain valuable insight into the public’s current state of awareness and understanding of PD, informing potential avenues for awareness campaigns and education initiatives. Potential confounding was mitigated through demographic subgroup analyses and by minimising measurement bias via piloting and standardised survey delivery.

## Results

### Description of sample

Altogether, the total number of members of the public who completed the survey was 796. Most participants were aged between 18 and 24 (31.7%), with smaller proportions in older age groups, particularly those over 65 (4.6%). The gender distribution was predominantly female (83.4%), with males comprising 16% of the sample. In terms of educational attainment, half (50%) of the respondents had completed secondary school, while 29.5% had completed an undergraduate degree. Experience with PD varied, with 36.1% reporting no experience, while 5.7% had extensive experience with the condition.

Characteristics of participants are described in Table 2:

**Table 2:** Characteristics of survey participants

Category	Subcategory	*N*	%
**Age Group**	18–24	252	31.7
	25–34	193	24.3
	35–44	119	14.9
	45–54	114	14.3
	55–64	81	10.2
	65+	37	4.6
**Gender**	Female	664	83.4
	Male	128	16
	Non-binary	2	0.3
	Prefer not to say	2	0.3
**Final/current level of education**	Secondary School	398	50
	Undergraduate	235	29.5
	Postgraduate	163	20.5
**Experience of Parkinson’s disease**	No experience	288	36.1
	Limited experience	203	25.5
	Some experience	167	21
	Moderate experience	93	11.7
	Extensive experience	45	5.7

### Knowledge of parkinson’s disease

Participant knowledge of PD varied significantly across different aspects of the condition. Awareness of PD as a neurological disorder was high, with 92.9% of respondents correctly identifying it as such. However, some misconceptions persisted, with 3.2% believing PD to be an autoimmune disease, 0.7% categorising it as cardiovascular, and 3.2% expressing uncertainty. Pearson chi-square tests indicated statistically significant associations between experience with PD and classification knowledge (χ² = 24.391, *p* = 0.018), as well as between age and understanding of PD as a medical condition (χ² = 25.158, *p* = 0.048), confirming the influence of age on PD categorisation. This shows that those of an older age, and those with more experience of PD are more likely to have a greater knowledge of the neurological nature of PD.

The genetic nature of PD was also a topic of divided perceptions. While 35.8% of participants believed PD is genetic, 30.7% disagreed, and 33.5% were uncertain. The Pearson chi-square (χ² = 42.864, *p* < 0.001) and likelihood ratio (45.885) revealed a significant relationship between age and beliefs about PD’s genetic nature, further supported by a linear trend in the linear-by-linear association (19.928, *p* < 0.001). We acknowledge that Q5 presents an oversimplified view of a complex condition, as PD involves both genetic and non-genetic factors. o Experience with PD also significantly influenced knowledge of its genetic nature (χ² = 17.583, *p* = 0.025), though this relationship was not linear (linear-by-linear association = 1.713, *p* = 0.191). These mixed findings reflect the broader scientific understanding of PD: while it is known that PD can have a genetic component, this is not always the case.

Participant recognition of PD symptoms was generally high, with tremor (97.4%), lack of balance (87.1%), and slow movement (83.3%) being the most commonly known motor symptoms. Speech difficulties (85.4%) and muscle rigidity (74.6%) were also well-recognised. Among non-motor symptoms, depression (68.6%), personality changes (63.6%), and loss of facial expression (63.7%) were identified by over 60% of participants, while chronic fatigue (55.5%) and drooling (51.9%) were less commonly recognised. The prevalence of these symptoms varied across age groups, with symptoms like shaking (χ² = 15.617, *p* = 0.008) and constipation (χ² = 12.196, *p* = 0.032) showing stronger associations with age. As experience with PD increased, awareness of symptoms such as muscle rigidity and urinary incontinence grew significantly, with chi-square values (e.g., urinary incontinence χ² = 114.238, *p* < 0.0001) reflecting notable differences in awareness based on experience. The data shows that while general recognition of symptoms is high, especially for motor symptoms like tremor and slow movement, awareness of non-motor symptoms varies, with greater experience leading to significantly better understanding of both common and lesser-known symptoms.

Awareness of PD treatments was reported by 42% of participants, while 45.2% were unaware, and 12.8% were uncertain. When asked about the potential for a cure, only 0.1% of respondents believed PD to have a cure, while 79.4% correctly reported that no cure exists, and a substantial portion (20.5%) answered “I don’t know”.

### Stigma and public perception

Stigma surrounding PD was witnessed by 33.7% of participants, while 39.9% had not encountered stigma, and 26.4% were unsure. Awareness of stigma increased with age, with 40.3% of participants aged 35–44, 38.6% of those aged 45–54, and 43.2% of individuals aged 65 + reporting exposure to stigma. The Pearson chi-square (χ² = 180.996, *p* < 0.001) demonstrated a strong association between age, experience with PD, and the perception of stigma. This shows overall that older participants, and those with more experience of the disease are more likely to have seen or experienced stigma related to PD.

When participants were asked about their primary concerns if diagnosed with PD, the two most common worries were the “fear of the future” (75.3%) and a “reduced ability to complete everyday tasks” (86.3%). Other concerns, such as “social isolation” (15.2%), “depression” (10.7%), “what others think of me” (5.5%), and “anxiety” (4%), were less frequently reported.

### Awareness and education

A significant majority of participants (89.1%) agreed that there was insufficient public awareness of PD in their community. The highest percentage of agreement (93.7%) came from the 18–24 age group. Regarding the best platforms to raise PD awareness, 46.2% of participants preferred social media, followed by television (15.5%) and schools (16.6%). Other platforms such as the internet (10%), doctor’s surgeries (5.5%), leaflets (3.3%), and newspapers (0.9%) were deemed less effective, with only 2% suggesting alternative methods for spreading awareness.

Responses for each question in the survey can be seen below in Table 3.

**Table 3:** Responses for all survey questions

Question	Response	*N*	%
**Q4. Have you heard of Parkinson’s disease?**	Yes	794	99.7
	No	2	0.3
	I don’t know	0	0
**Q5. Do you know anyone personally who has been diagnosed with Parkinson’s disease?**	Yes	442	55.5
	No	346	43.4
	I don’t know	8	1.1
**Q6. How would you rate your knowledge of Parkinson’s disease?**	Poor	294	36.9
	Fair	178	22.4
	Average	199	25
	Good	105	13.2
	Excellent	20	2.5
**Q7. What type of disease do you think Parkinson’s disease is?**	Cardiovascular	6	0.7
	Respiratory	0	0
	Neurological	740	92.9
	Gastrointestinal	0	0
	Autoimmune	26	3.2
	Endocrine	0	0
	I don’t know	24	3.2
**Q8. Do you think Parkinson’s disease is genetic?**	Yes	285	35.8
	No	244	30.7
	I don’t know	267	33.5
**Q9. What age do you think people are first diagnosed with Parkinson’s disease?**	45 years or younger	48	6.1
	45–55 years old	146	18.3
	55–65 years old	197	24.7
	Over 65 years old	101	12.7
	No particular age group	269	33.8
	I don’t know	35	4.4
**Q10. Could you please choose any of the following symptoms that could be associated with Parkinson’s disease?**	Shaking (tremor)	776	97.4
	Lack of balance	693	87.1
	Speech difficulties	680	85.4
	Slow movement	663	83.3
	Muscle rigidity	594	74.6
	Depression	546	68.6
	Loss of facial expression	507	63.7
	Personality changes	506	63.6
	Chronic fatigue	442	55.5
	Drooling	413	51.9
	Urinary incontinence	346	43.5
	Constipation	276	34.7
**Q11. Are you aware of any treatments available for Parkinson’s disease?**	Yes	334	42
	No	360	45.2
	I don’t know	102	12.8
**Q12. Does Parkinson’s disease have a cure?**	Yes	1	0.1
	No	632	79.4
	I don’t know	163	20.5
**Q13. Have you ever witnessed stigma or misconceptions related to Parkinson’s disease?**	Yes	268	33.7
	No	318	39.9
	I am not sure	210	26.4
**Q14. If you were to develop Parkinson’s disease**,** which two matters would you worry about the most from the following?**	Fear of the future	599	75.3
	Depression	85	10.7
	Anxiety	32	4
	Social isolation	121	15.2
	What others think of me	44	5.5
	Reduced ability to complete everyday tasks	687	86.3
**Q15. Do you think there is enough awareness about Parkinson’s disease in your community?**	Yes	17	2.1
	No	709	89.1
	I don’t know	70	8.8
**Q16. Where would be most beneficial to present educational information about Parkinson’s disease to raise awareness in your community?**	Internet	80	10
	Social media	368	46.2
	Newspapers	7	0.9
	Television	123	15.5
	Doctor’s surgery	44	5.5
	Leaflets	26	3.3
	Schools	132	16.6
	Other	16	2

## Discussion

This study provides valuable insights into public knowledge and perceptions of PD across Ireland, emphasising demographic influences and key gaps in understanding.

The findings of this study are consistent with previous research examining public knowledge and awareness of PD, including a comprehensive scoping review that identified gaps in understanding regarding the disease’s symptoms, aetiology, and treatment options [4]. For example, several studies highlighted that public knowledge of motor symptoms was higher than non-motor symptoms [17, 21, 22], and two studies revealed a lack of knowledge of medication for PD [17, 23]. The review also shed light on prevailing public attitudes, particularly the association between PD and psychological challenges such as depression and social isolation, alongside the notable scarcity of accessible educational resources aimed at improving public awareness. The present study reinforces these observations, underscoring the continued lack of public understanding of PD and the knowledge surrounding its clinical presentation and management.

While recognition of PD as a neurological disorder, and awareness of motor symptoms such as tremor and slow movement is high in this study, non-motor symptoms like chronic fatigue remain less recognised than motor symptoms, reflecting broader gaps in public health literacy. For example, studies show public awareness of dementia [24] and multiple sclerosis [25] often focuses on visible symptoms, with limited understanding of underlying disease characteristics.

Similar trends are observed in studies on diabetes mellitus, where knowledge gaps exist concerning risk factors and general awareness [26]. In the context of neurological disorders, studies assessing public understanding of conditions like cerebral palsy in Saudi Arabia [27] and motor neuron disease in Pakistan [28] also reveals significant deficits in public knowledge, particularly in the areas of causes, symptoms, and diagnosis. This aligns closely with the findings of this study, underscoring the widespread nature of these knowledge gaps across various conditions. Addressing these issues through targeted education and public health initiatives is essential to enhance overall understanding and reduce misconceptions.

The primary concerns expressed by participants in this study, fear of the future and reduced ability to perform everyday tasks, are reflective of broader anxieties commonly reported across other conditions including cancer, COPD and epilepsy. One study [29] specifically explored the anxieties of people with diverse chronic illnesses such as epilepsy, cancer and COPD and found them to have similar fears to those with PD. These fears often stem from uncertainties about disease progression, potential loss of autonomy, and the impact on QoL. Social isolation, depression, and anxiety, while reported less frequently, also mirror emotional and psychological challenges noted in chronic illness literature [29–31]. Addressing these fears through targeted public education could play a pivotal role in alleviating anxiety and fostering resilience. By providing clear information about disease management, available support systems, and coping strategies, educational initiatives can empower individuals and help reduce the fear associated with a PD diagnosis, ultimately promoting a more informed and supportive community.

Stigma remains a pervasive and detrimental issue for individuals living with PD, with a notable proportion participants in this study reporting they have experiences of societal stigma. These findings align with a recent systematic review focused specifically on stigma in PD [7], which highlighted stereotypes and misconceptions about the disease. Key contributors to stigma identified in the review included the duration since diagnosis and, more prominently, a lack of public awareness and inadequate media representation. Furthermore, the review emphasised the psychological consequences of stigma, including increased feelings of shame, reduced self-esteem, and social withdrawal, as well as the various coping mechanisms adopted by individuals with PD to navigate these challenges. The parallels between this study and the review are evident, particularly in highlighting both the presence of stigma and the critical gap in public education regarding PD. These patterns are not unique to PD, they reflect broader trends observed in other chronic and neurological conditions such as epilepsy and MND, where misinformation and social stigma exacerbate emotional distress and social marginalisation [32–36]. Additionally, it has been acknowledged that Q13 (“Have you ever witnessed stigma or misconceptions related to Parkinson’s disease?”) is subjective and may be interpreted variably due to its broad phrasing. However, it was included to explore public awareness of stigma, which, even if reported anecdotally, provides valuable insight into how PD is perceived in everyday social contexts.

The study highlights the critical role of social media as a powerful platform for raising awareness about PD particularly among younger demographics. This shift toward digital health communication reflects the growing reliance on social media for disseminating health information. Social media’s unique ability to share valuable information rapidly and widely makes it an effective tool for public health campaigns. Evidence from multiple studies supports its success in increasing awareness of conditions like dementia [37, 38], where one study found a social media campaign on pain in dementia to have increased public awareness. This demonstrates the capacity of social media to engage diverse audiences and foster greater understanding [39–41]. By leveraging social media, public health initiatives can combat stigma, enhance knowledge, and reach broader, more varied populations efficiently.

### Strengths and limitations

A key strength of this study is its comprehensive analysis of public knowledge and perceptions of PD across the island of Ireland, providing valuable insights into demographic trends and specific knowledge gaps. By examining age, education, and personal experience, the study highlights how awareness varies across groups, helping identify where targeted education and awareness efforts are most needed. This study is the first of its kind to explore the topic using an all-Ireland perspective. The large representation of younger adults and females offers a unique opportunity to tailor future awareness campaigns to these groups, potentially enhancing the effectiveness of public health initiatives. However, this demographic skew also represents a limitation, as it may not fully capture the perspectives of older adults and males, who are also important stakeholders in PD awareness and education. The study did not capture wider demographic factors such as occupation or ethnicity, which may influence perceptions of PD. Cultural attitudes in Ireland toward ageing and illness can affect understanding and engagement with awareness campaigns. Additionally, the reliance on self-reported data could introduce bias, as participants’ knowledge and perceptions may not accurately reflect the general population. Recruitment through Parkinson’s charities may have introduced bias by attracting participants more familiar with the condition. However, this was balanced by broader outreach via social media and other non-condition-specific charities, helping to capture a wider cross-section of the general population. Despite these limitations, the study provides a strong foundation for developing targeted educational interventions to improve public understanding of PD.

## Conclusion

This study emphasises the urgent need for targeted public health interventions to address persistent knowledge gaps and misconceptions about PD. By focusing on education tailored to demographic variations and leveraging the power of digital platforms, particularly social media, public health campaigns can significantly enhance understanding, reduce stigma, and alleviate the anxieties associated with a PD diagnosis. Addressing both motor and non-motor symptom awareness, along with combating stigma and fostering resilience through clear communication and support systems, will be pivotal in improving the overall public perception and management of PD. These findings contribute to a broader understanding of the challenges faced across various chronic conditions, highlighting the importance of a comprehensive, informed, and empathetic approach in public health discourse.

## Electronic supplementary material

Below is the link to the electronic supplementary material.

Supplementary Material 1

Supplementary Material 2

## Data Availability

Some of the data generated or analysed during this study are included in this published article and its supplementary information files; additional datasets are available from the corresponding author on reasonable request.

## Abbreviations

PD: Parkinson’s Disease
QoL: Quality of Life
PI: Parkinson’s Ireland
ROI: Republic of Ireland
NI: Northern Ireland
COPD: Chronic Obstructive Pulmonary Disease
