# Supplementary material for: Understanding the public knowledge and awareness of parkinson’s disease in Ireland: a cross-sectional survey
Source: BMC Public Health. 2025 Jul 3;25:2339. doi: 10.1186/s12889-025-23573-8 (PMC12224858; doi:10.1186/s12889-025-23573-8)
Supplement: Supplementary file 2 — Supplementary Material 2 [file 12889_2025_23573_MOESM2_ESM.docx]

**A questionnaire about the public awareness and perceptions of Parkinson’s disease.**

**Demographic Information**

1. Age:

18-24 25-34 35-44 45-54 55-64 65+

1. Final Education:

Secondary school Undergraduate Postgraduate

1. Experience of Parkinson’s disease:

No experience Limited experience Some experience

Moderate experience Extensive Experience

**Awareness and Knowledge**

1. Have you heard of Parkinson’s disease before taking this survey?

Yes No

1. Do you know anyone personally who has been diagnosed with Parkinson’s disease?

Yes No

1. How would you rate your knowledge of Parkinson’s disease?

No knowledge at all Not very knowledgeable Somewhat knowledgeable

Very knowledgeable

1. What type of disease do you think Parkinson’s disease is?

Cardiovascular Respiratory Neurological Gastrointestinal

Autoimmune Endocrine

1. Do you think Parkinson’s disease is genetic?

Yes No

1. What age do you think people are first diagnosed with Parkinson’s disease?

45 years or younger 45-55 years old 55-65 years old

Over 65 years old No particular age group I don’t know

1. Can you please choose any of the following symptoms that could be associated with Parkinson’s disease?

Shaking (tremor) Lack of balance Muscle rigidity

Slow movement Speech difficulties Loss of facial expression

Constipation Urinary incontinence Drooling

Chronic fatigue Personality changes Depression

**Treatment**

1. Are you aware of any treatments available for Parkinson’s disease?

Yes No

1. Does Parkinson’s disease have a cure?

Yes No

**Stigma and Misconceptions**

1. Have you ever witnessed stigma or misconceptions related to Parkinson’s disease?

Yes No

1. If you were to develop Parkinson’s disease, which two matters would you worry about the most from the following?

Fear of the future Depression Anxiety

Social isolation What others think of me

Reduced ability to complete everyday tasks

**Support and Awareness**

1. Do you think there is enough awareness about Parkinson’s disease in your community?

Yes No

1. Where would be most beneficial to present educational information about Parkinson’s disease to raise awareness in your community?

Internet Social Media Newspapers Television

Doctors’ surgery Leaflets Schools Other

**Thank you for taking part in this survey.**
